# A specialized unit for women with schizophrenia: Results from the healthcare model Observatories-Monitoring Stations and Interventions.

**DOI:** 10.1192/j.eurpsy.2024.597

**Published:** 2024-08-27

**Authors:** J. P. Paolini San Miguel, M. Natividad, M. V. Seeman, E. Román, A. Balagué, B. Palacios, N. Bagué, E. Izquierdo, E. Rial, J. A. Monreal, A. González Rodríguez

**Affiliations:** ^1^Mental Health, Mutua Terrassa University Hospital. University of Barcelona, Terrassa, Spain; ^2^Psychiatry, University of Toronto, Toronto, Canada; ^3^Clinical and Health Psychology, Autonomous University of the State of Morelos, Cuernavaca, Mexico

## Abstract

**Introduction:**

There are many theoretical reasons to implement gender-specific care for schizophrenia. For all these reasons, the Mutua Terrassa-Functional Unit for Women with Schizophrenia was inaugurated in January 2023 in the context of a community mental health service.

**Objectives:**

Our aim today is to describe the health care model applied in this newly initiated unit.

**Methods:**

We created a healthcare model in our new unit consisting of A)Five observatories of Health (somatic morbi-mortality, hyperprolactinemia-HPRL, substance use disorders, social exclusion/discrimination, and drug safety); B)Monitoring stations or vigilance teams (reflecting the 5 observatories); and C)resulting actions (specific interventions). The observatory teams each meet monthly. In this presentation, according to the healthcare model we implemented, we first describe data about the original patient recruitment and then focus on the observatories of somatic morbi-mortality and hyperprolactinemia.

**Results:**

From 265 potentially eligible women, 42 were included in the 5 observatories. (A) of the 11 women in the observatory of somatic morbi-mortality, 10 women had died within the last 24 months. Causes of Death: (1)respiratory tract disease (n=5,45.4%), (2)cancer (n=3;27.3%): lung cancer (n=1), pancreatic cancer (n=1), kidney cancer (n=1), (3)ischemic colitis (n=1;9%), (4)Alzheimer disease (n=1;9%). 2) Morbidity. One woman had an ongoing glioblastoma. (B)Observatory of HPRL. Eight women with moderate/severe HPRL were included. Strategies for lowering prolactin levels were discussed with neuroendocrinologists. Interventions:adjunctive aripiprazole (n=3), switch to aripiprazole (n=2), lowering antipsychotic doses (n=2), and adjunctive cabergoline (n=1).

**Conclusions:**

Designating special teams to focus on specific problems of women with schizophrenia will reduce morbidity and improve outcomes in this vulnerable population.

**Disclosure of Interest:**

None Declared

